# Antiradical, Chelating and Antioxidant Activities of Hydroxamic Acids and Hydroxyureas

**DOI:** 10.3390/molecules16086232

**Published:** 2011-07-25

**Authors:** Marijana Zovko Končić, Monika Barbarić, Ivana Perković, Branka Zorc

**Affiliations:** Faculty of Pharmacy and Biochemistry, University of Zagreb, A. Kovačića 1, Zagreb HR-10000, Croatia; Email: mbarbaric@pharma.hr (M.B.); iperkovic@pharma.hr (I.P.); bzbz@pharma.hr (B.Z.)

**Keywords:** hydroxamic acids, hydroxyureas, free radical, oxidative stress, reactive oxygen species

## Abstract

Reactive oxygen species, along with reactive nitrogen species, may play an important role in the pathogenesis and progress of many diseases, including cancer, diabetes and sickle cell disease. It has been postulated that hydroxyurea, one of the main treatments in sickle cell disease, achieves its activity partly also through its antioxidant properties. A series of hydroxyurea derivatives of L- and D-amino acid amides and cycloalkyl-*N*-aryl-hydroxamic acids was synthesized and investigated for their radical scavenging activity, chelating properties and antioxidant activity. All the compounds showed exceptional antiradical activities. For example, free radical scavenging activities of investigated hydroxyureas were higher than the activity of standard antioxidant, butylated hydroxyanisole (BHA). Moreover, most of the investigated hydroxamic acids were stronger Fe^2+^ ion chelators than quercetin. In addition, the investigated compounds, especially hydroxamic acids, were proven to be excellent antioxidants. They were as effective as BHA in inhibiting β-carotene-linoleic acid coupled oxidation. It is reasonable to assume that the antioxidant activity of the investigated compounds could contribute to their previously proven biological properties as cytostatic and antiviral agents.

## 1. Introduction

A growing body of evidence suggests that endo- and exogenous reactive oxygen species (ROS) may have an important role in the expansion and progression of tumors. In addition, the research has shown that the various types of cancer examined to date exhibit an imbalance in their antioxidant mechanisms. In the near future new insights in cancer therapies, based on modulation of cellular redox status, may lead the way to additional tools against carcinogenesis induced by ROS [1]. Oxidative stress is an imbalanced state of oxidants and antioxidants in organism, initiated by endogenous ROS, such as superoxide anion and hydrogen peroxide. These moderately strong oxidants can, in reaction with substances such as nitric oxide (NO) or transition metals, be converted into species with pronounced reactivity such as peroxynitrite and hydroxyl radicals. Those are molecules capable of attacking sensitive cellular targets like lipids, proteins and nucleic acids causing their inhibition and accelerated degradation. Thus, oxidative stress inflicts multiple levels of cellular damage, which propagates a vicious cycle. These consequences of oxidative stress construct the molecular basis in the development of many diseases. One of the ROS-mediated causes of cancer is gene mutations (modification of pyridine and purine bases) and post-translational modifications leading to disruption of cellular processes [2]. Therefore, in the last decade antioxidant compounds have received increased attention from medical researchers for their potential activities in preventing cancer, cardiovascular disorders, as well as aging. Analysis of activities of new antioxidant compounds of synthetic and natural origin have been subject of several publications [3,4,5].

One of the diseases where oxidative stress is especially pronounced is sickle cell disease (SCD). Today, hydroxyurea is considered by most physicians in the developing world as the treatment of choice for severe SCD. It seems that hydroxyurea exerts its activity through several mechanisms such as promotion of increase of fetal hemoglobin level, erythrocyte alterations, myelosuppression, and others [6]. In addition, it seems that some of its biological activity is achieved by ameliorating the damage caused by oxidative stress. Overproduction of ROS by enzymatic and non-enzymatic pathways promotes intravascular oxidative stress that can disrupt NO homeostasis [7]. However, hydroxyurea can be oxidized by heme groups to produce NO *in vitro*. Thus, the NO-donor properties of hydroxyurea may contribute to its beneficial effects in SCD [6].

In our previous papers, synthesis of hydroxyureas and hydroxamic acids with cytostatic and antiviral activities was described [8,9]. Thus, in an attempt to further study potential beneficial effects of those substances, their antioxidant activity was thoroughly investigated and compared.

## 2. Results and Discussion

**Figure 1:**
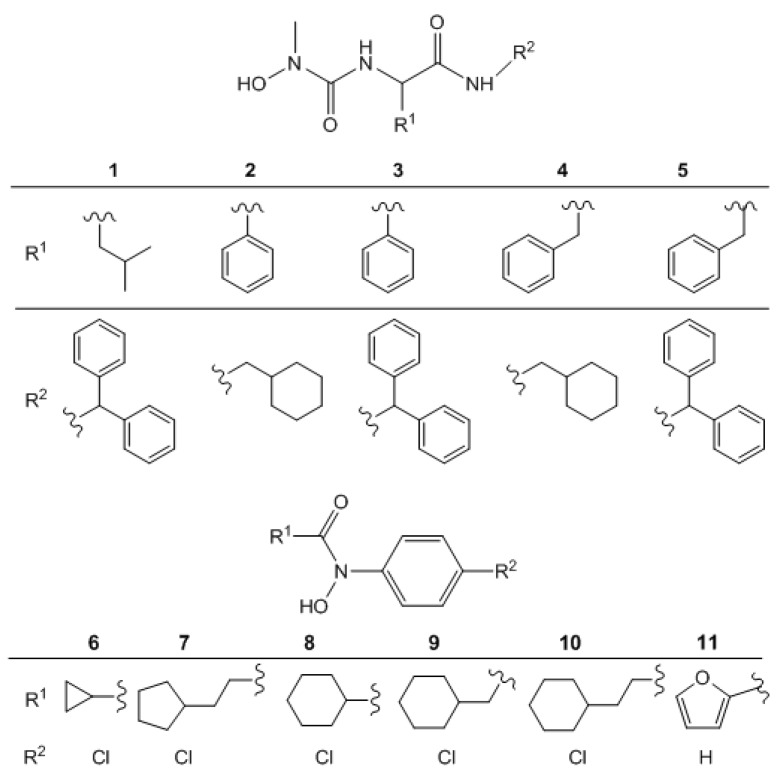
Hydroxyureas **1**–**5** and hydroxamic acids **6**–**11** investigated in this study.

Hydroxyurea derivatives of L- and D-amino acid amides **1**–**5** and cycloalkyl-*N*-arylhydroxamic acids **6**–**11** were synthesized and their structures were confirmed as described in literature. All analytical and spectral data were in agreement with the previously published data [8,9,10]. The structures of the synthesized compounds are presented in Figure 1.

### 2.1. DPPH Radical Scavenging Activity

At high concentrations, free radicals are mediators of damage to cell structures, nucleic acids, lipids and proteins. Examples of ROS-induced DNA damage products involve single- or double-stranded DNA breaks, purine, pyrimidine, or deoxyribose modifications, and DNA cross-links. Such modification of genetic material may represent the first step in mutagenesis, carcinogenesis and ageing. Free radical-mediated DNA damage has been found in various cancer tissues. Thus, it has been hypothesized that the free radical scavengers are molecules that could prevent or limit the damage provoked by free radicals [11,12]. Capability of hydroxamic acids and hydroxyureas to scavenge free radicals has been assessed in reaction with DPPH, a relatively stable free radical.

**Figure 2:**
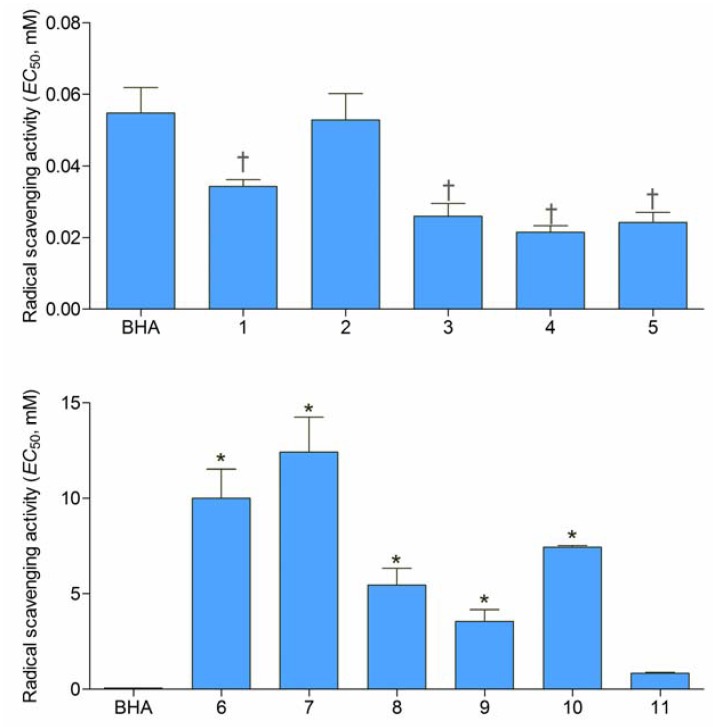
Radical scavenging activities of hydroxyureas **1**–**5** and hydroxamic acids **6**–**11** expressed as *EC*_50_ (means ± SD, *n* = 3). Statistically significant differences: * less active than BHA; ^†^ more active than BHA (*P* < 0.05).

DPPH, in its radical form, has strong visible absorption and high molar extinction coefficient at 517 nm. Upon reaction with an antioxidant the absorbance diminishes [13]. Investigated hydroxyureas **1**–**5** demonstrated remarkable radical scavenging activity (Figure 2) with *EC*_50_ values lower than 0.06 mM. Moreover, with the exception of **2**, all the hydroxyureas showed activity statistically higher than the activity of synthetic antioxidant BHA. Such exceptional activity was not surprising because a previous investigation of primaquine derivatives indicated that introduction of hydroxyureas in the molecule had high positive impact on the reactivity towards DPPH free radical [14]. Hydroxamic acids in this study were less reactive towards DPPH free radical than hydroxyureas (Figure 2). Compounds **6**–**10** displayed relatively weak free radical scavenging activity in comparison to BHA. Despite the fact that halogenation increases free radical scavenging activity of some compounds [15] the most potent hydroxamic acid in this study was the only non-halogenated derivative **11** with activity statistically equal to the activity of BHA. This might be explained by the proximity of aromatic furane ring to the hydroxamic moiety and possibility of dislocation of unpaired electron formed in reaction with DPPH radical. Activity of the substances was not in correlation with their log *P* values (Table 1).

**Table 1:** Log *P* and reactivity in β-carotene-linoleate assay.

Compound	Log *P*	*ANT* (%) ^a,b^	*AA-60* (%) ^a,c^
**1**	3.12	62.45 ± 5.55 *	63.46 ± 3.01 *
**2**	2.30	64.47 ± 5.55 *	65.72 ± 3.06 *
**3**	3.23	60.67 ± 5.43 *	63.15 ± 3.37 *
**4**	2.46	60.66 ± 1.38 *	62.80 ± 0.89 *
**5**	3.41	67.33 ± 4.71 *	69.93 ± 2.84 *
**6**	2.02	72.73 ± 2.45 *	73.76 ± 1.14
**7**	3.79	89.13 ± 1.89 ^†^	88.55 ± 0.92
**8**	3.34	88.61 ± 1.74 ^†^	88.03 ± 1.15
**9**	3.82	94.39 ± 1.03 ^†^	94.22 ± 1.19
**10**	4.25	89.86 ± 0.97 ^†^	89.54 ± 1.80 ^†^
**11**	1.53	54.96 ± 4.67 *	57.43 ± 2.53 *
BHA	*n.c* *.*	79.59 ± 2.06	80.55 ± 1.43

^a^ means ± SD (*n* = 3); ^b^ antioxidant activity; ^c^ normalized antioxidant activity at 60-min of incubation; *n.c.*: not calculated; Statistically significant differences within columns: * less active than BHA; ^†^ more active than BHA (*P* < 0.05).

### 2.2. Chelating Activity

**Figure 3:**
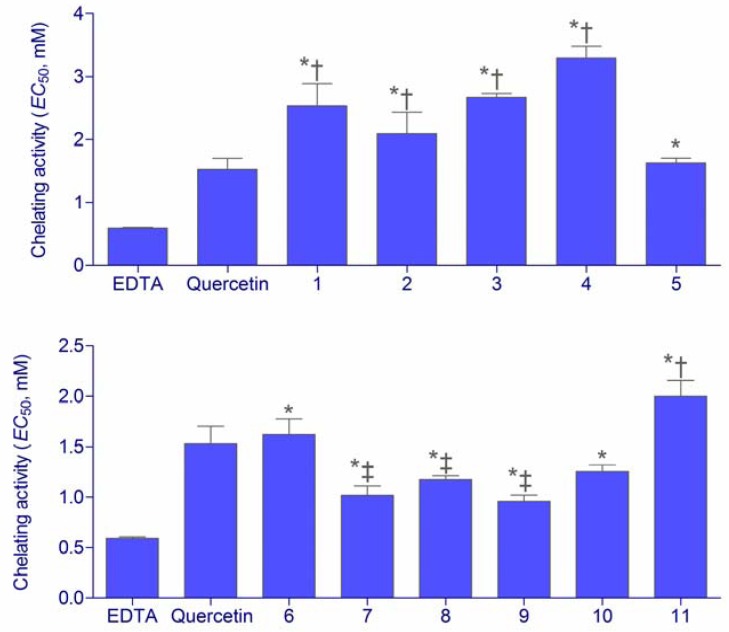
Fe^2+^ chelating activities of hydroxyureas **1**–**5** and hydroxamic acids **6**–**11** expressed as *EC*_50_ (means ± SD, *n* = 3). Statistically significant differences: * less active than EDTA; ^†^ less active than quercetin; ^‡^ more active than quercetin (*P* < 0.05).

Direct reaction of a substance is not the only mechanism by which the antioxidants may display their activity. Secondary, preventive, or type 2, antioxidants act through numerous possible mechanisms. These antioxidants do not convert free radicals to more stable products but slow the rate of oxidation by several different mechanisms. One of the most important mechanisms of action of secondary antioxidants is chelation of prooxidant metals. Iron and other transition metals (copper, chromium, cobalt, vanadium, cadmium, arsenic, nickel) promote oxidation by acting as catalysts of free radical reactions. These redox-active transition metals transfer single electrons during changes in oxidation states. Chelation of metals by certain compounds decreases their prooxidant effect by reducing their redox potentials and stabilizing the oxidized form of the metal. Chelating compounds may also sterically hinder formation of the metal hydroperoxide complex [16]. Chelating activity of hydroxamic acids and hydroxyureas was compared to two chelating standards, EDTA and quercetin (Figure 3).

All the investigated substances were capable of chelating Fe^2+^ ions. The metal chelating effects of the samples were dependent on concentration and linearly increased with the sample concentration increase. The affinity of hydroxyureas **1**–**4** for ferrous ions was relatively low in comparison to quercetin and EDTA. However the activity of hydroxyurea **5** was the same as the activity of quercetin. On the other hand, hydroxamic acids **6**–**11** investigated in this assay were stronger chelating agents than hydroxyureas. Although somewhat weaker chelators than EDTA, they demonstrated relatively high activity in comparison to quercetin. The chelating activity of substance **11** was lower than quercetin, while the other hydroxamic acids were either equally active (**6**, **10**) or even even stronger (**7**–**9**) ferrous ion chelators. Therapeutically, the ion chelating activity of drugs may be especially important in diseases that include extensive hemolysis or frequent blood transfusions, such as SCD. For example, deferoxamine, deferasirox and other iron chelators have been shown efficient in treatment of iron overload caused by blood transfusions in SCD [17]. Thus, excellent activity of the investigated hydroxamic acids may also implicate the use of the investigated compounds as iron chelators, similarly to some other compounds of that class [17,18].

### 2.3. β-Carotene Linoleic Acid Assay

**Figure 4:**
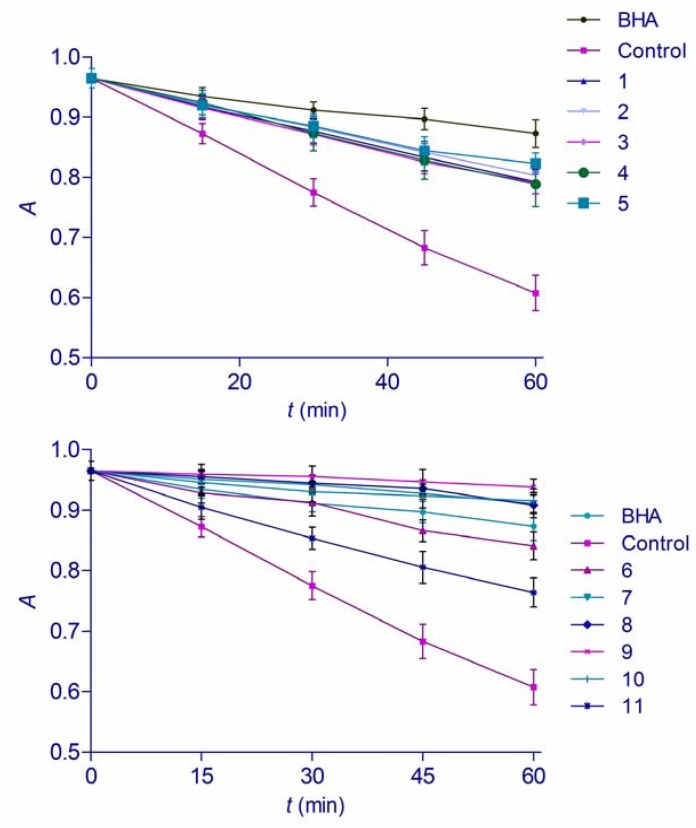
Bleaching of emulsion with β-carotene in presence of hydroxyureas **1**–**5** and hydroxamic acids **6**–**11** (means ± SD, *n* = 3). Absorbance was measured at *λ* = 470 nm.

Oxidation of an aqueous emulsion of β-carotene and linoleic acid was employed as a test for measuring total antioxidant activity of the hydroxamic acids and hydroxyureas. In this particular model, heat induces formation of linoleic acid free radical. The radical then reacts with conjugated double bonds of β-carotene, causing a rapid degradation and discoloration [19]. Thus, by simulation of the oxidation of the membrane lipid components in the presence of antioxidants, this test gives an insight of the inhibitory effect of substances on the lipid peroxidation. It also measures the capacity to inhibit the formation of conjugated diene hydroperoxide arising from linoleic acid oxidation [20]. The presence of an antioxidant can reduce the extent of β-carotene destruction by reacting with the linoleate free radical or any other free radical formed within the system. The substances investigated in this study were able to reduce the rate of degradation of β-carotene significantly in comparison with the control (Figure 4). Antioxidant activity was measured as a percentage of inhibition of lipid peroxidation (*ANT*) (Table 1). Hydroxyureas **1**–**5** demonstrated relatively low antioxidant activity in comparison with BHA. Hydroxamic acids **6**–**11**, on the other hand, displayed outstanding antioxidant properties in this assay. Not only was their *ANT* value statistically higher than the activity of investigated hydroxyureas, but most of them (**7**–**10**) were also more potent antioxidants than BHA. According to Amarowicz *et al.* [19], the antioxidant activity expressed as the percent of inhibition of coupled oxidation of β-carotene and linoleic acid against the water and BHA control samples, based on absolute changes of absorbance at distinct points in time during the assay (*AA-60*), rather than as an average rate, provides another way of evaluating antioxidant activity in β-carotene-linoleic acid assay. In present study, if antioxidant activity was evaluated as *AA-60* (Table 1), the activities of compounds **6**–**9** were statistically equal to that of BHA while **10** was more potent antioxidant. Hydroxamic acids have been previously shown as potent lipid peroxidation inhibitors [21]. Moreover, it has been proven that the introduction of hydroxamic moiety into some organic acids creates potent antioxidants, capable of hindering linoleic acid degradation in a similar manner as butylated hydroxytoluene, one of the antioxidant used by food industry [22]. Outstanding protective effect of hydroxamic acids towards heat-induced linoleic acid oxidation found in this study confirms those findings. Both, *ANT* and *AA-60* values of hydroxamic acids and log *P* correlated linearly with *r*^2^ values of 0.87. This high correlation coefficient could be explained by better diffusion of lipophilic compounds into micelles with linoleic acid and β-carotene where they can exhibit their antioxidant activity.

## 3. Experimental

### 3.1. General

Melting points were determined on a Stuart Melting Point Apparatus SMP3 (Stuart Barworld Scientific, UK) and are uncorrected. IR spectra were recorded on a FTIR Perkin Elmer Paragon 500 spectrometer (Perkin Elmer, UK). ^1^H- and ^13^C-NMR spectra were recorded on a Varian Gemini 300 spectrometer (Varian, USA), operating at 300 and 75.5 MHz for the ^1^H and ^13^C nuclei, respectively. For absorbance measurements, a Stat Fax 3200 (Awareness Technologies, USA) microplate reader was used.

### 3.2. Chemicals

Hydroxyureas, namely *N*-benzhydryl-2-(*N’*-methyl-*N’*-hydroxyureido)-L-4-methylpentanamide (*N’*-methyl-*N’*-hydroxycarbamoyl-L-leucine benzhydrylamide) (**1**), *N*-cyclohexanemethyl-2-(*N’*-methyl-*N’*-hydroxyureido)-D-2-phenylethamide (*N’*-methyl-*N’*-hydroxycarbamoyl-D-phenylglycine cyclohexanemethylamide) (**2**), *N*-benzhydryl-2-(*N’*-methyl-*N’*-hydroxyureido)-D-2-phenylethamide (*N’*-methyl-*N’*-hydroxycarbamoyl-D-phenylglycine benzhydrylamide) (**3**), *N*-cyclohexanemethyl-2-(*N’*-methyl-*N’*-hydroxyureido)-L-3-phenylpropanamide (*N’*-methyl-*N’*-hydroxycarbamoyl-L-phenylalanine cyclohexanemethylamide) (**4**), *N*-benzhydryl-2-(*N’*-methyl-*N’*-hydroxyureido)-L-3-phenylpropanamide (*N’*-methyl-*N’*-hydroxycarbamoyl-L-phenylalanine benzhydrylamide) (**5**) and the following hydroxamic acids: *N*-(4-chlorophenyl)-*N*-hydroxycyclopropanecarboxamide (**6**), *N*-(4-chlorophenyl)-3-cyclo- pentyl-*N*-hydroxypropanamide (**7**), *N*-(4-chlorophenyl)-*N*-hydroxycyclohexanecarboxamide (**8**), *N*-(4-chlorophenyl)-2-cyclohexyl-*N*-hydroxyacetamide (**9**) and *N*-(4-chlorophenyl)-3-cyclohexyl-*N*-hydroxypropanamide (**10**) were synthesized according to our published procedures [8,9]. *N*-phenyl-2-furohydroxamic (**11**) was prepared following reference [5]. All analytical and spectral data were in agreement with the previously published data [8,9,10]. Butylated hydroxyanisole (BHA), 2,2-diphenyl-1-picrylhydrazyl (DPPH), β-carotene, linoleic acid and ferrozine were purchased from Sigma-Aldrich (USA). Other chemicals and solvents used were of analytical grade.

### 3.3. Antiradical Activity

Free radical scavenging activity (*RSA*) was evaluated by the scavenging of DPPH radicals as described by Yen and Chen [23] with some modifications. Methanolic solution of DPPH (20 μL, 0.735 mg/mL) was added to 200 μL of either ethanolic solution of the test sample or methanol (negative control). The mixture was vortexed for 1 min and then left to stand at room temperature in the dark. After 30 min absorbance was read at 545 nm. BHA was used as a positive control. *RSA* for DPPH free radical was calculated using *A*_cont_ (absorbance of the negative control, e.g., blank solution without test compound) and *A*_sample_ (absorbance of the substance solution). *RSA* was expressed as the concentration that scavenges 50% of DPPH free radicals (*EC*_50_).

### 3.4. Fe^2+^ Chelating Activity

The chelating activity (*ChA*) of the investigated substances toward ferrous ions was studied as described in reference [24]. To an aliquot of the methanolic solution of the test substance (150 μL), 0.25 mM FeCl_2_ solution (50 μL) was added. After 5 min, the reaction was initiated by adding 1.0 mM ferrozine solution (100 μL). Absorbance at 545 nm was recorded after 10 min of incubation at room temperature. A reaction mixture containing methanol (150 μL) instead of substance solution served as a control. Quercetin and EDTA were used as the chelating standards. *ChA* was calculated using *A*_cont_ (absorbance of the negative control, e.g., blank solution without test compound) and *A*_sample_ (absorbance of the substance solution). Chelating activity was expressed as *EC*_50_, the concentration that chelates 50% of Fe^2+^ ions.

### 3.5. β-Carotene-linoleic Acid Assay

The antioxidant activity of the substances was evaluated using the β-carotene-linoleic acid system according to modified literature procedures [19,24]. Tween 40 (200 mg) and β-carotene solution in chloroform (1.0 mL, *γ* = 0.2 g/L) were mixed. After removing chloroform in a rotary evaporator, linoleic acid (20 mg) and aerated distilled water (30 mL) were added to the oily residue with vigorous stirring. Aliquots (200 μL) of thus obtained emulsion were added to sample solution in methanol (50 μL, 0.02 mM). A reaction mixture containing methanol (50 μL) instead of sample solution served as a control. BHA was used as an antioxidant standard. After adding the emulsion to the sample solution, the reaction mixture was incubated at 50 °C for 1 h. During that period, the absorbance was measured at 450 nm at 15-minute intervals, starting immediately after sample preparation (*t* = 0 min) until the end of the experiment (*t* = 60 min). The percent of antioxidant activity (*ANT*) was calculated using *R*_cont_ and *R*_sample_, average bleaching rates of the water control and antioxidant (test compound or BHA), respectively. In addition, antioxidant activity was calculated from the absolute changes in absorbance at *t* = 60 min and was expressed as *AA-60* value as described by Amarowicz *et al*. [19].

### 3.6. Statistical and Log P Analysis

All assays were performed in triplicate. The results were expressed as mean ± SD. Statistical comparisons were made using one-way ANOVA, followed by Dunnett’s post-hoc test for multiple comparisons with the control and Student’s *t*-test with Welch correction for comparison of two groups of compounds. *P* values <0.05 were considered statistically significant. Statistical analyses were performed using the JMP V6 from SAS software (SAS Institute, Cary, NC, USA). Log *P* values were calculated at Virtual Computational Chemistry Laboratory (VCCLAB, http://www.vcclab.org, 2005) [25].

## 4. Conclusions

In the present study, radical scavenging, metal chelating and antioxidant activities of several hydroxyureas and hydroxamic acids were investigated by scavenging effect on the DPPH free radical, metal chelation effect in the Fe^2+^-ferrozin test system, as well as by β-carotene-linoleic acid assay. Hydroxyureas investigated in this study were excellent radical scavengers, with activity similar to that of BHA. On the other hand, hydroxamic acids were more successful in chelation of ferrous ions. They also demonstrated pronounced antioxidant activities in the prevention of heat-induced oxidation of linoleic acid. The results of this study imply that antioxidant activity of investigated compounds may significantly contribute to their cytostatic activity. In addition, similarly to some other compounds of the two classes, investigated hydroxyureas and hydroxamic acids may also have potential in treatment of SCD due to their antioxidant, antiradical and chelating properties.
